# Type D personality is associated with impaired psychological status and unhealthy lifestyle in Icelandic cardiac patients: A cross-sectional study

**DOI:** 10.1186/1471-2458-12-42

**Published:** 2012-01-18

**Authors:** Erla Svansdottir, Krista C van den Broek, Hrobjartur D Karlsson, Thorarinn Gudnason, Johan Denollet

**Affiliations:** 1 CoRPS--Center of Research on Psychology in Somatic diseases, P.O. Box 90153, Tilburg University, 5000 LE Tilburg, The Netherlands; 2 Icelandic Heart Association, Holtasmári 1, 201 Kópavogur, Iceland; 3 Landspitali-University Hospital, Landspítali Hringbraut, 101 Reykjavík, Iceland

**Keywords:** Coronary heart disease, Health-related behavior, Personality, Psychological, Risk factors

## Abstract

**Background:**

Type D (distressed) personality has been associated with adverse cardiac prognosis and poor emotional well-being in cardiac patients, but it is still unclear what mechanisms link Type D personality with poor clinical outcomes in cardiac patients. In the present cohort of Icelandic cardiac patients, we examined potential pathways that may explain this relationship. The objectives were to examine 1) the association between Type D personality and impaired psychological status, and to explore whether this association is independent of disease severity; and 2) the association between Type D personality and an unhealthy lifestyle.

**Methods:**

A sample of 268 Icelandic coronary angiography patients (74% males (N = 199); mean age 62.9 years (SD 10.5), range 28-85 years) completed the Type D Scale (DS14), Hospital Anxiety and Depression Scale (HADS), and Perceived Stress Scale (PSS) at hospitalization. Health-related behaviors were assessed 4 months following angiography. Clinical data were collected from medical files.

**Results:**

Type D personality was associated with an increased risk of anxiety (OR 2.97, 95% CI:1.55-5.69), depression (OR 4.01, 95% CI:1.42-11.29), and stress (OR 5.99, 95% CI:3.08-11.63), independent of demographic variables and disease severity. Furthermore, fish consumption was lower among Type Ds, as 21% of Type Ds versus 5% of non-Type Ds consumed fish < 1 a week (*p *< 0.001). Type D patients were also more likely to smoke at follow-up (22% versus 10%, *p *= 0.024) and to use antidepressants (17% versus 9%, *p *= 0.049) and sleeping pills (49% versus 33%, *p *= 0.019) compared to non-Type Ds. Type D personality was not associated with other health-related behaviors, aside from trends towards less fruit and vegetable consumption, and more weight gain.

**Conclusion:**

Type D personality was associated with psychological distress and an unhealthy lifestyle in Icelandic cardiac patients. Future studies should further investigate the association between Type D personality and health-related behaviors.

## Background

Evidence linking psychological factors with adverse prognosis in patients suffering from cardiovascular disease (CVD) has accumulated in recent years [1,2]. In this realm of research, one specific personality construct, the Type D (distressed) personality, has shown particular promise as a potential risk factor for poor prognosis in CVD patients. Type D personality refers to high scores on two stable personality traits, negative affectivity (NA) and social inhibition (SI), and portrays individuals who frequently experience negative emotions (elevated NA), but tend to inhibit emotional expression due to fear of rejection (elevated SI) [3]. This combination of elevated negative affect and high social inhibition is thought to have a negative impact on clinical outcomes in cardiac patients, rather than one of the two personality traits alone [4].

The Type D personality concept was originally developed to identify cardiac patients at risk of developing emotional and interpersonal difficulties [3,5], and has as such, been strongly associated with psychological comorbidity in cardiac patients, e.g. post-traumatic stress disorder [6], anxiety [7-9], depression [9,10], and vital exhaustion [11]. However, further research revealed that Type D personality is also associated with increased morbidity [12], mortality [13-15] and a poor quality of life [16] across diverse CVD patient groups, where it has been associated with a 3-fold increased risk of poor prognosis [17]. Several studies have demonstrated that the effect of Type D personality on adverse outcomes is independent of biomedical risk factors, such as hypertension [14,16] and disease severity, comprised by multi-vessel disease and left ventrical function [14,18].

With a prevalence of 25-38% in cardiac patients [14,19-21], Type D personality can potentially influence the prognosis and well-being of a substantial number of cardiac patients.

It is still unclear what mechanisms link Type D personality with poor clinical outcomes in CVD patients [22]. Mediating mechanisms may include both physiological and behavioral factors [13,23,24]. Recent findings have suggested that negative health-related behavior [22] and inadequate consultation behavior [25] may play a role in the behavioral factor vicinity. Individuals experiencing psychological distress may for instance be more prone to use maladaptive coping styles, such as increased smoking and poor diet [26], which again can negatively impact the disease process. Further investigations on how Type D personality influences the health of cardiac patients are needed, especially since such analysis would provide clues for possible targets for intervention in these patients [13,14]. In addition, there is also a need for more cross-cultural investigations on Type D personality and its influence, since previous investigations have primarily focused on samples of Belgian and Dutch origin [27].

The aim for the present study was twofold: (1) to investigate the relationship of Type D personality with anxiety, depression and stress in Icelandic cardiac patients, and to explore whether this association is independent of indicators of disease severity; and (2) to investigate the relationship of Type D personality with certain health-related behaviors in these patients.

## Methods

### Participants

The original participant sample consisted of 315 patients who underwent a coronary angiography at Landspitali-University hospital from January to May 2008. These patients were a part of a larger study, the "Risk factors, prognosis and success of medical procedures in patients undergoing coronary angiography at Landspitali-University Hospital", and were included in the current study because they answered additional questionnaires measuring anxiety, depression and stress at baseline. Participants were first approached when hospitalized to the coronary care unit or upon arrival to the emergency ward. Follow-up assessments were administered with a phone call to participants in July 2008, approximately four months after discharge (M = 106 days, (SD 27.2 days)). A total of 268 patients (85%) completed the follow-up, and were included in the final study sample. Of the excluded 47 patients (15%), six patients (2%) were deceased, one patient lived abroad and was therefore not included in follow-up, three individuals (1%) refused to participate in the follow-up and the remaining 37 (11%) could not be reached. The follow-up group did not differ in age from the patient group not reached at follow-up (M = 62.8 years, (SD 10.5) versus M = 65.4 years (SD 9.3), t(313) = 1.56, *p *= 0.12). The study protocol was approved by the medical ethics committee of The National Bioethics Committee in Iceland. The study was conducted conform to the ethical tenets developed by the World Medical Association, as espoused in the Declaration of Helsinki. All patients provided written informed consent.

### Demographics

Information concerning gender and age was gathered from medical records, while data concerning educational level (elementary, higher education (secondary or university)) and family status (living alone/widowed, married/living with partner) were collected by self-report from participants.

### Clinical variables measured at baseline

Information regarding disease classification, traditional coronary artery disease (CAD) risk factors, and disease severity were retrieved from patients' medical records. Information on disease status was classified as follows: CAD, myocardial infarction (MI), arrhythmias, heart valve disease and heart failure. Traditional CAD risk factors were defined in the following way: smoking (yes, no); hypertension (no hypertension treatment, current hypertension treatment); on blood-lipid lowering medication (no, yes); diabetes (no, yes); and overweight (body mass index, BMI). Disease severity was defined by a) the number of coronary arteries affected by CAD (0 or 1 artery versus ≥ 2 arteries), and b) cardiac history (previous percutaneous coronary intervention (PCI), previous MI, and/or a previous coronary artery bypass surgery (CABG)).

### Measures

All participants were administered the Icelandic versions of the Type D scale (DS14) [19], the Hospital Anxiety and Depression Scale (HADS) [28], and the Perceived Stress Scale (PSS) [29] at baseline, when hospitalized for a coronary angiography.

The DS14 comprises two seven-item subscales (NA and SI) in order to measure the tendency to experience negative emotions (NA, "I am often irritated") and the tendency to inhibit self-expression in social interactions (SI, "I am a closed kind of person"), the two components of Type D personality. The report answer format is on a Likert scale ranging from 0 (false) to 4 (true). Total scores on both subscales range from 0 to 28. Participants were defined as having Type D personality if they scored ≥ 10 on both subscales [19]. A recent study using item-response theory has shown the cut of ≥ 10 to be the best to distinguish between Type D and non-Type D individuals [30]. Results from factor analyses on the scale have indicated a clear two factor structure, representing negative affectivity and social inhibition [19,31,32]. The Icelandic version of the DS14 has good internal consistency (Cronbach's α = 0.87-0.88 for NA; Chronbach's α = 0.84-0.85 for SI) and psychometric evaluations have supported the construct validity of the scale [33].

The HADS measures symptoms of anxiety and depression and was specifically developed and tested in physically ill people [28]. This questionnaire contains seven items for each mood status. Participants answer on a four-point scale (0-3) how well each statement refers to them, and total scores for each domain range from 0 to 21. The Icelandic version of the HADS identifies symptoms of depression and anxiety sufficiently well [34], and reliability estimates across various studies range from 0.78-0.86 for anxiety and 0.65-0.85 for depression [35]. Continuous scores on the HADS were used for the main analysis and dichotomous scores were used for a logistic regression. Depression and anxiety scores on HADS were categorized in a similar way as recommended by the authors, with the exception that borderline symptoms and full symptoms were pooled into one category, such that scores ≥ 8 indicated presence of symptoms of anxiety and depression.

The PSS is a 14-item questionnaire which measures perceived stress [29], more specifically, the degree to which situations in one's life are appraised as stressful. Items include questions such as "In the last month, how often have you felt nervous and stressed" and "In the last month, how often have you felt that you were unable to control the important things in your life?" Responses are measured on a five-point Likert scale ranging from 0 (never) to 4 (very often), and the total score ranges from 0 to 56. The PSS has good psychometric properties [29,36]. The Icelandic version of the PSS has comparable psychometric properties to the original version (Davíðsdóttir S, Bachman T, [Association of stress and gender with health and health-habits], Bachelor's thesis, University of Iceland, 1991), with reliability coefficients of α = 0.89 in a healthy sample and α = 0.90 in a patient sample (Svansdóttir E, [Translation and psychometric evaluation of the DS-14 scale among university students and heart patients], cand. psych thesis, University of Iceland, 2006). In the current study, continuous scores were used for the main analysis. To indicate heightened symptoms of perceived stress, we used a cut-off score at the 75th percentile.

### Health-related behaviors measured at four-month follow-up

Assessment of health-related behaviors was conducted four-months after discharge, by a phone call by a researcher to participants, where standard questions regarding exercise, diet, smoking and psychopharmacological medication use were administered. Specific questions included: a) whether patients engaged in sufficient exercise per week (> 20 min 3× a week); b) whether patients had gained weight after discharge (yes, no); c) rehabilitation attendance after discharge (yes, no); d) whether they had breakfast every morning (yes, no); e) daily consumption of fruits and vegetables (not daily, daily); f) frequency of fish consumption (< once per week, weekly); g) smoking (yes, no); and h) regular use of sleeping pills, antidepressants, and/or anxiety-reducing medication.

### Statistical analyses

Prior to analysis, missing values on the DS14, HADS and PSS were replaced if the number of missing items per participant did not exceed three on the DS14 and HADS subscales, or four for the total PSS scale. Missing items were replaced with each participant's average score on the subscale the missing items belonged to. For each scale, replaced missing items were ≤ 1% of the total number of items. Four patients (1.5%) did not complete the HADS scale adequately and were excluded from all analysis that included HADS scores. Sixteen patients (6%) had ≥ four items missing on the PSS scale, and were excluded from analysis involving PSS scores.

Differences in demographics, clinical variables and health-related behavior between Type D and non-Type D individuals were explored with chi-square calculations for nominal variables and independent t-tests for continuous variables. Independent t-tests were administered to examine basic differences in anxiety, depression and stress scores between Type D and non-Type D patients. The association of Type D personality with anxiety, depression and perceived stress was assessed with multiple linear regressions. Each separate model was implemented with a hierarchical entry, where Type D personality was inserted at the first step, while age, gender, disease severity, cardiac history, education and family status were added in the second step as covariates. Two outliers were identified in the anxiety and depression models and excluded from further analysis in those models. Underlying assumptions were inspected for each regression model and indicated no problems. The effect size for differences in anxiety, depression, and perceived stress scores by Type D personality were estimated using Cohen's d calculations. Linear regression analysis was used to test the unique and shared predictive power of both Type D components for anxiety, depression and stress, where continuous NA and SI scale scores (0-28) were included as predictors instead of Type D personality. Each linear regression model was run twice, first with NA inserted at the first step and SI at the second step, and then with SI inserted at the first step and NA at the second step, in order to assess the unique explained variance of NA and SI. Furthermore, a logistic regression, which incorporated the same covariates as the linear regression, was conducted to assess the odds ratio associated with Type D patients for manifestation of increased symptoms of anxiety, depression, and stress. For this analysis, all predictors were inserted into the model simultaneously using the enter method.

Finally, a re-analysis was conducted for all significant associations where the Type D/non-Type D categorization (≥ 10 on NA and SI) was substituted with continuous NA and SI scale scores [37]. Inter-quartile ranges were used to rescale NA and SI scores and the NA by SI interaction term, so that a one unit difference represented a clinically relevant metric. Within these NA inter-quartile distribution, 70% (N = 49) of Type Ds fell within the 4th quartile, and 30% (N = 21) within the 3rd quartile. For SI 49% (N = 34) of Type Ds fell within the 4th quartile and 51% (N = 36) within the 3rd quartile. In the inter-quartile NA by SI scores, 89% (N = 62) of Type Ds were within the 4th quartile and 11% (N = 8) within the 3rd quartile. Linear regression models for anxiety, depression, and stress were re-executed, with NA, SI and the NA by SI interaction term entered at the first step and covariates at the second. For health-related risk markers, binary logistic regression analyses (stepwise procedure) were used with NA, SI and the NA by SI interaction term as predictors.

All analyses were two-tailed and alpha < 0.05 was used to indicate statistical significance. The SPSS 17 statistical software for windows was used for the analysis (Statistical Package for Social Sciences, Chicago, IL, USA).

## Results

### Demographical and clinical variables

Mean age in the sample was 62.9 years (SD 10.5) and males were more prevalent (74%, N = 199) than females. A total of 26% of patients were defined as having Type D personality, which is in line with previous research [3,19-21]. Baseline characteristics of Type D and non-Type D individuals are presented in Table 1. Type D patients were on average younger than non-Type D patients, but no differences emerged in gender distribution, family status or educational level between groups. Likewise, prevalence of traditional CAD risk factors was similar across groups, except that Type D patients were less likely to be on hypertension treatment compared to their non-Type D counterparts. No difference was found in disease severity, as measured by the number of vessels affected by CAD, nor previous cardiac history (former PCI, MI and/or CABG) between Type D and non-Type D participants.

**Table 1:** Differences in demographical and clinical variables between Type D and non-Type D patients*

	Total (N = 268)	non-Type D (N = 198)	Type D (N = 70)	*p*-value
**Demographics**				

Age Mean (SD)	62.9 (10.5)	63.6 (10.6)	60.7 (10.1)	0.045

Male	74% (199)	73% (146)	27% (53)	0.75
	
Female	26% (69)	75% (52)	25% (17)	

Widowed/Living alone	22% (59)	21% (42)	24% (17)	0.59

Elementary education (N = 267)	39% (105)	38% (75)	43% (30)	0.48

**Disease**				

Coronary artery disease	69% (186)	69% (136)	71% (50)	
	
Myocardial infarction	11% (30)	12% (24)	9% (6)	
	
Arrhythmia	4% (10)	3% (5)	7% (5)	
	
Heart valve disease	4% (12)	5% (10)	3% (2)	
	
Heart failure	2% (4)	2% (3)	1% (1)	
	
Unspecified chest pain/other	10% (26)	10% (20)	9% (6)	
	
CAD risk factors and disease severity**				

Hypertension treatment	60% (157)	65% (126)	45% (31)	0.004

High blood-lipids treatment	65% (170)	67% (129)	60% (41)	0.36

Diabetes	11% (29)	11% (22)	10% (7)	0.80

Current smoking (baseline)	22% (59)	19% (38)	30% (21)	0.067

BMI Mean (SD)	28.9 (5.0)	28.9 (4.9)	28.7 (5.1)	0.70

≥ 2 Vessel disease	39% (105)	39% (78)	39% (27)	0.90

Previous PCI, MI or CABG	30% (80)	33% (64)	23% (16)	0.13

* Data are presented as percentages (N) unless otherwise specified

** Due to missing values N varies between 262 and 268 patients

### The association of Type D personality with anxiety, depression and stress

**Figure 1:**
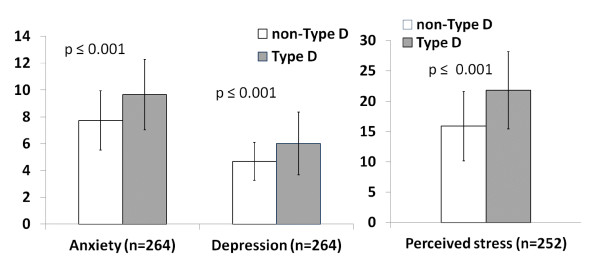
**Differences in average anxiety, depression and stress scores by Type D personality (with 95% confidence intervals)**.

Type D patients had significantly higher anxiety, depression and perceived stress scores compared to their non-Type D counterparts (M = 9.7 (SD 2.6) versus M = 7.7 (SD 2.2), t(262) = 5.92, *p *< 0.001 for anxiety; M = 6.0 (SD 2.3) versus M = 4.7 (SD 1.4), t(86.2) = 5.54, *p *< 0.001 for depression; M = 21.8 (SD 6.4) versus M = 15.9 (SD 5.7), t(250) = 7.02, *p *< 0.001 for perceived stress; see Figure 1). Further analysis with multiple linear regressions showed that the association between Type D personality and higher scores on anxiety, depression and perceived stress was independent of age, gender, family status, education, disease severity and cardiac history. In all cases, Type D personality had a strong association at the first step, (explaining 13%, 11% and 16% of variance in anxiety, depression and stress scores, respectively), and the association remained when covariates were inserted into the model at the second step. The inclusion of covariates contributed to a 6% increase in explained variance of anxiety scores, but did not significantly improve model fit for depression or perceived stress (see Table 2). The effect sizes associated with Type D personality were high (Cohen's d = 0.78, 0.74, and 0.93 for anxiety, depression, and perceived stress respectively).

**Table 2:** Multiple linear regression of anxiety, depression and perceived stress scores by Type D personality and covariates

	Anxiety (N = 256)			Depression (N = 256)			Perceived stress (N = 244)		
	B	β	R^2^	B	B	R^2^	B	β	R^2^
*Step 1*			0.13			0.11			0.16
Type D personality	1.88	.36**		1.23	.33**		5.76	.41**	
*Step 2*			ΔR^2 ^= 0.06, *p *= 0.010			ΔR^2 ^= 0.04, *p *= 0.074			ΔR^2 ^= 0.03, *p *= 0.22
Type D personality	1.74	.33**		1.19	.32**		5.54	.39**	
Age	-0.05	-.21**		0.01	.04		-0.09	-.15*	
Gender (female)	0.80	.15*		-0.63	-.17*		0.51	.04	
Family status (married)	0.29	.05		-0.39	-.10		-0.33	-.02	
Higher education	-0.18	-.04		-.45	-.13*		-1.24	-.10	
≥ 2 Vessel disease	-0.02	-.00		-0.28	-.08		0.27	.02	
Previous cardiac history	-0.06	-.01		-0.10	-.03		0.83	.06	

*p ≤ 0.05; **p ≤ 0.001

Analysis of the unique and shared predictive power of both Type D subcomponents revealed that the association between Type D and anxiety was primarily driven by NA (31% of the variance), while the total variance explained by both factors was 34%. SI did not significantly contribute to this model of anxiety; the shared variance of both factors was 2.5%. Conversely, both NA and SI contributed to the association with depression and perceived stress, with 9% shared variance for both measures. The unique effect of NA was larger in both cases, with NA and SI explaining 9% and 4% of depression scores and 17% and 1% in perceived stress scores, respectively.

Multivariate logistic regression analyses replicated these findings, indicating that Type D patients had about three to four times greater odds of experiencing some symptoms of anxiety (OR 2.97, 95% CI:1.55-5.69, *p *< 0.001) and depression (OR 4.01, 95% CI:1.42-11.29, *p *= 0.009), and nearly six times greater odds of heightened perceived stress (OR 5.99, 95% CI:3.08-11.63, *p *< 0.001) compared to non-Type D patients, independent of covariates.

### Health-related behavior

A comparison of health-related behavior four-months after discharge between groups indicated that diet, medication use and smoking may differ between Type D and non-Type D patients (Table 3). First of all, fish consumption was considerably less frequent among Type D patients. A total of 21% of Type D's consumed fish less than once a week compared to only 5% of non-Type D's (χ ^2^_(1, N = 268) _= 16.40; *p *< 0.001). A trend towards less consumption of fruits and vegetables in Type D patients was found as well, but 81% of non-Type Ds versus 70% of Type Ds consumed fruits and vegetables on a daily bases (χ^2^_(1, N = 267) _= 3.44, *p *= 0.064). However, no differences were found regarding whether patients had breakfast every day. Likewise, no difference was found in exercise between groups. A trend towards Type D patients being more likely to have gained weight after the angiography compared to non-Type D patients was found although not significant (χ^2^_(1, N = 268) _= 3.37; *p *= 0.066). A separate analysis was conducted post hoc in overweight patients (BMI ≥ 25) to explore this matter further, and revealed that 18% of overweight Type D patients reported having gained weight after the angiography compared to 8% of non-Type D patients (χ^2^_(1, N = 221) _= 4.47; *p *= 0.035).

**Table 3:** Prevalence of certain health-related behavior practices across groups at follow-up*

	N	Total	non-Type D	Type D	*p*-value
Exercise					
Minimal exercise (< 20 min 3× a week)	268	11% (29)	11% (21)	11% (8)	0.85

Have attended rehabilitation	263	30% (78)	28% (55)	33% (23)	0.43

**Weight**					

Gained weight after the angiography	268	11% (30)	9% (18)	17% (12)	0.066

Gained weight (obese patients, BMI ≥ 25)	221	10% (23)	8% (13)	18% (10)	0.035

**Diet**					

Have breakfast every day	267	90% (240)	91% (179)	87% (61)	0.38

Consume fruits and vegetables every day	267	78% (208)	81% (159)	70% (49)	0.064

Consume fish seldom (≤ 1 a week)	268	9% (25)	5% (10)	21% (15)	0.001

**Smoking**					

Smoking prevalence at follow-up	266	10% (27)	8% (15)	17% (12)	0.024

**Psychopharmacological medication use**					

Use sleeping pills regularly	261	37% (96)	33% (63)	49% (33)	0.019

Use antidepressants regularly	263	11% (29)	9% (17)	17% (12)	0.049

Use anxiety-reducing medication regularly	263	12% (32)	11% (22)	15% (10)	0.49

*Data are presented as percentages (N) unless otherwise specified

At follow-up, the prevalence of smoking was 17% in Type D patients versus 8% in non-Type D patients (χ^2^_(1, N = 266) _= 5.09; *p *= 0.024). A similar trend for smoking was noted at baseline, although not statistically significant. Finally, more patients with Type D personality reported use of antidepressants (17% versus 9%; χ^2^_(1, N = 263) _= 3.86; *p *= 0.049) and sleeping pills (49% versus 33%; χ^2^_(1, N = 261) _= 5.46; *p *= 0.019), compared to their non-Type D counterparts. However, no difference was found in reported anxiety medication use between groups.

### Secondary analysis of significant results using re-scaled Type D scale scores

NA was a significant predictor for anxiety (b = 0.65, *p *< 0.001), depression (b = 0.30, *p *= 0.006), and stress (b = 0.36, *p *= 0.001), and SI was a significant predictor for depression (b = 0.25, *p *= 0.009). After adjustment for these NA and SI main effects, the interaction term of NA by SI was not significant in these analyses of anxiety, depression and stress. In binary logistic regression models of health-related behaviors, the NA by SI interaction term was associated with higher odds of smoking at follow-up (OR 1.50, 95% CI: 1.01-2.21, *p *= 0.04) and less fish consumption (OR 0.48, 95% CI: 0.31-0.74, *p *= 0.001), and NA with more use of antidepressant medications (OR 1.89, 95% CI: 1.29-2.77, *p *= 0.001). No association was found between NA, SI, or NA by SI with weight gain (in patients with BMI ≥ 25) or use of sleep medication.

## Discussion

This study investigated the association between Type D personality and psychological distress in Icelandic cardiac patients, explored whether the association was confounded by indicators of disease severity, and examined the relationship between Type D personality and certain health-related behaviors. As expected, patients with Type D personality had a worse psychological status compared to their non-Type D counterparts, which was independent of the patient's demographic status and markers of disease severity. These results are in support for the notion that Type D personality is associated with impaired psychological well-being in cardiac patients, and are congruent with previous findings [8,9], where Type D has been associated with a three-fold risk of increased psychological distress [17]. Further analysis revealed that the association between Type D and poor psychological status was mainly driven by NA, but that SI also had a significant unique contribution to depression and perceived stress. NA and SI shared considerable variance in depression/stress scores, indicating the effect of Type D personality. Other researchers also found that the interaction of NA and SI predicted increased stress levels [24].

Regarding health-related behaviors, Type D patients displayed a lower prevalence of fish consumption and a trend towards less fruit and vegetable consumption compared to non-Type Ds, as well as a predisposition to smoke at follow-up and a higher prevalence of sleep- and antidepressant medication use. The higher prevalence of psychopharmacological medication use has been noticed previously, where post-MI patients with a Type D personality were significantly more likely to use benzodiazepines as compared to non-Type D patients [38], and provides further support to the current findings that Type D individuals experience more symptoms of anxiety, depression and stress.

The finding that Type D personality was not associated with indicators of disease severity is in line with previous findings [14,18,39], providing further evidence that Type D personality is not related to disease severity. Other studies also found no association between psychological factors and extent of coronary atherosclerosis [40]. Hence, the adverse effects of Type D personality on cardiac prognosis may be mediated through other pathways, such as behavioral and physiological factors [13,23,24].

Apart from the unexpected link between Type D and a lower prevalence of hypertension treatment, no association was found with the traditional CAD risk factors. This fits well with the general consensus that the influence of Type D on cardiac health is not mediated through biomedical risk factors [14,16]. The lower hypertension treatment prevalence could be due to a poorer self-management in Type D patients, as Type D has previously been associated with poor medication adherence [41]. Type D individuals are also less likely to seek appropriate medical care [42] or to have regular medical check-ups [22].

Re-analyses of significant associations of Type D personality with outcome variables, using continuous NA, SI, and NA by SI scores confirmed the association of the NA and SI subscales with depression, and the main effects of NA with anxiety and stress. The NA by SI interaction term was not significant in these analyses, probably as the main effects of the Type D subcomponents were already accounted for. Regarding health-behaviors, the NA by SI interaction term was associated with more smoking and lower fish consumption and NA with use of antidepressant medications. These findings suggest that categorical and dimensional definitions of Type D personality are not necessarily mutually exclusive, but represent two different ways of capturing the psychological profiles of individuals [43].

Behavioral processes are believed to constitute one of the main mediating mechanisms linking personality and psychological distress with impaired health [44] and increased CVD risk [23,26], and recent findings suggest that health-related behavior may explain 40% of the association between personality traits and mortality [45]. Thus, management of behavioral processes such as health-related behavior may be crucial to reduce distress-related CVD risk [23]. The current findings suggested that some important aspects of health-related behavior may differ between Type D and non-Type D patients. A distinct difference in fish consumption was found, and previously, Type D personality has been associated with less sensible diet in healthy individuals [22]. Healthy diet choices are considered an important part of CVD risk reduction [46], where for example increased consumption of fruits and vegetables [47,48], fish, and reduced intake of fried foods [46,47] are recommended. Unhealthy diet has been associated with an increased risk of acute myocardial infarction worldwide, and is estimated to account for nearly 30% of the population attributable risk [47]. Thus, a predisposition towards unhealthy diet choices is a plausible mediating mechanism in the relationship between Type D personality and clinical CVD events, and as such, should be inspected more thoroughly.

Although no differences in exercise and rehabilitation were found, a trend towards a higher prevalence of weight gain was noticed in Type D patients, which was significant in a post hoc analysis in overweight patients. Hence, overweight Type D patients may be more prone to gain weight after a coronary angiography, although how much weight these participants gained is unknown. Weight loss is of significant importance in obese individuals, and cardiac patients in particular, as it can improve or prevent many of the obesity-related risk factors for coronary heart diseases [49].

Type D patients were more likely to smoke at follow-up than non-Type D patients, although this difference was not significant at baseline. Other studies have also found an association between Type D personality and smoking [33,50,51]. Difficulties with maintaining smoking abstinence have been related to neuroticism [52,53] and psychological distress [52]. Type D individuals may experience the prospect of smoke-cessation as a more threatening and stressful event, due to their tendency to experience things in a more negative way, and might therefore need more support with altering their smoking habits. Taken together, Type D individuals may need more assistance with smoking cessation and other health-related behaviors, such as changing dietary habits. Behavioral interventions to reduce psychological distress might facilitate more successful modifications of unhealthy lifestyles [54].

Assessment of Type D personality could be useful to identify patients who have an increased risk of adverse clinical events [17]. Type D personality has also been associated with inadequate consultation behavior [25,55], poor medication adherence [41] and negative illness perception [56] in cardiac patients. Rozanski [57] has argued that cardiologists should consider including a brief screening of psychological factors that might influence patient behavior and adherence into their standard care. The DS14 is a short, reliable measure of Type D that is easy to administer (2-3 minutes) and score [19], and that could be used by health professionals to identify Type D patients that may benefit from more tailored intervention in clinical care.

Little is known about the population attributable risk Type D poses for CVD incidence in the community, as the main emphasizes in Type D research has not been to assert causal connection with CVD incidence, but rather to examine the association between general distress and prognosis in cardiovascular populations [17]. As a consequence, most studies on Type D personality and coronary heart disease have been conducted in cardiovascular samples. Yet, a number of general population studies on Type D personality have exposed Type D personality as a vulnerability factor for worse self-reported health status, more somatic health complaints and disease-promoting mechanism [58] and unhealthier lifestyle behaviors [22].

Finally, the current findings support the cross-cultural validity for the association of Type D personality with psychological distress, and are consistent with recent finding from Denmark [59], Germany [60] and the United States [61]. Thus, the effect of Type D personality is not limited to Dutch and Belgian populations [9].

The results of the current study should be interpreted with some caution due to the following limitations. The participant sample consisted of a heterogeneous group of cardiac patients undergoing coronary angiography, and thus measurements of disease severity employed in this study may not portray effectively worse disease status for a small proportion of the sample (for instance in arrhythmia patients). In addition, the current findings regarding Type D and psychological status might be susceptible for reverse causation, due to the cross-sectional origin, but previous longitudinal reports demonstrating that Type D predicts onset, prevalence and severity of psychological distress after adjustments for baseline depression [17] diminish such a risk. Furthermore, health-related behaviors were assessed with self-report and not by extensive and psychometrically examined measurement devices. Yet, the current sample represented a broad group of cardiac patients undergoing a coronary angiography in the only hospital in Iceland that performs angiographies, and thus the sample portrays effectively the population of cardiac patients of a whole nation as non-selectively as possible.

## Conclusions

In summary, the results of the present study indicate that Type D personality is associated with more psychological distress and unhealthy lifestyle behaviors in Icelandic cardiac patients, and support the cross-cultural validity of the Type D personality construct. Further studies should be implemented to investigate, in more detail, the association between Type D personality and health-related behavior, for such investigations could generate intervention strategies to improve the prognostic outlook for cardiac patients with Type D personality.

## Competing interests

The authors declare that they have no competing interests.

## Authors' contributions

All authors have contributed significantly to the paper, with the following specifications. ES is the lead author of the manuscript. Her work included data analysis and interpretation, as well as drafting and revising the manuscript. HDK and ÞG lead the design and coordination of the study, and helped with drafting and reviewing the manuscript. JD and KCB provided substantial contribution to the analysis and interpretation of data, and provided assistance with drafting and reviewing the manuscript. All authors have read and approved the final manuscript.

## Pre-publication history

The pre-publication history for this paper can be accessed here:

http://www.biomedcentral.com/1471-2458/12/42/prepub
